# Twelve-year trends in the prevalence and risk factors of diabetes and prediabetes in Turkish adults

**DOI:** 10.1007/s10654-013-9771-5

**Published:** 2013-02-14

**Authors:** Ilhan Satman, Beyhan Omer, Yildiz Tutuncu, Sibel Kalaca, Selda Gedik, Nevin Dinccag, Kubilay Karsidag, Sema Genc, Aysegul Telci, Bulent Canbaz, Fulya Turker, Temel Yilmaz, Bekir Cakir, Jaakko Tuomilehto

**Affiliations:** 1 Division of Endocrinology and Metabolism, Department of Internal Medicine, Istanbul Faculty of Medicine, Istanbul University, Turgut Ozal Caddesi, Capa, 34093 Istanbul, Turkey; 2 Department of Clinical Biochemistry, Istanbul Faculty of Medicine, Istanbul University, 34093 Istanbul, Turkey; 3 Department of Public Health, Marmara University Faculty of Medicine, 34668 Istanbul, Turkey; 4 Division of Endocrinology and Metabolism, Department of Internal Medicine, Yildirim Beyazit University Medical Faculty, 06800 Ankara, Turkey; 5 South Ostrobothnia Central Hospital, 60220 Seinäjoki, Finland; 6 Centre for Vascular Prevention, Danube University, 3500 Krems, Austria; 7 Division of Endocrinology and Metabolism, Department of Internal Medicine, Istanbul Faculty of Medicine, Istanbul University, 34093 Istanbul, Turkey

**Keywords:** Diabetes mellitus, Impaired fasting glucose, Impaired glucose tolerance, Obesity, Hypertension, Clinical epidemiology

## Abstract

There is concern about an emerging diabetes epidemic in Turkey. We aimed to determine the prevalence of diagnosed and undiagnosed diabetes, prediabetes and their 12-year trends and to identify risk factors for diabetes in the adult Turkish population. A cross-sectional, population-based survey, ‘TURDEP-II’ included 26,499 randomly sampled adults aged ≥ 20 years (response rate: 87 %). Fasting glucose and biochemical parameters were measured in all; then a OGTT was performed to identify diabetes and prediabetes in eligible participants. The prevalence of diabetes was 16.5 % (new 7.5 %), translating to 6.5 million adults with diabetes in Turkey. It was higher in women than men (*p* = 0.008). The age-standardized prevalence to the TURDEP-I population (performed in 1997–98) was 13.7 % (if same diagnostic definition was applied diabetes prevalence is calculated 11.4 %). The prevalence of isolated-IFG and impaired glucose tolerance (IGT), and combined prediabetes was 14.7, 7.9, and 8.2 %, respectively; and that of obesity 36 % and hypertension 31.4 %. Compared to TURDEP-I; the rate of increase for diabetes: 90 %, IGT: 106 %, obesity: 40 % and central obesity: 35 %, but hypertension decreased by 11 % during the last 12 years. In women age, waist, body mass index (BMI), hypertension, low education, and living environment; in men age, BMI, and hypertension were independently associated with an increased prevalence of diabetes. In women current smoking, and in men being single were associated with a reduced risk. These results from one of the largest nationally representative surveys carried out so far show that diabetes has rapidly become a major public health challenge in Turkey. The figures are alarming and underscore the urgent need for national programs to prevent diabetes, to manage the illness and thus prevent complications.

**Electronic supplementary material:**

The online version of this article (doi:10.1007/s10654-013-9771-5) contains supplementary material, which is available to authorized users.

## Background

The new diabetes atlas published recently indicated that diabetes is increasing worldwide [1]. Following rapid economic growth, increase in life expectancy, and changes in lifestyle [2, 3], diabetes becomes one of the major public health issues also in Turkey [1, 4, 5]. With diverse health challenges, health authorities in Turkey need robust data on the epidemiology and impact of diabetes in order to plan and prioritize their health programs.

A cross-sectional survey, TURDEP-I [6] conducted in 1997–1998, comprising a nationally representative sample of 24,788 adult Turkish people (aged ≥ 20 years) showed that the prevalence of diabetes was 7.2 % and that of impaired glucose tolerance (IGT) 6.7 %. Although these were higher than previous reported estimates [7, 8], direct comparison between studies was difficult due to methodological differences [6–8]. Furthermore, the prevalence of diabetes and prediabetes was probably underestimated because an oral glucose tolerance test (OGTT) was not performed in all participants. Yet, it is well-known that isolated post-challenge hyperglycemia is common in many populations [9, 10].

Twelve years after the first survey, ‘The Turkish Epidemiology Survey of Diabetes, Hypertension, Obesity and Endocrine Disease (TURDEP-II)’ was conducted in the same study centers as TURDEP-I during 15 January to 11 June 2010. This cross-sectional survey was designed to provide current and reliable data on the 12-year trends in the prevalence of diabetes, prediabetes, and associated metabolic risk factors in the adult population of Turkey.

## Methods

### Study centers

Because considerable differences exist among regions in Turkey [11], we included samples from both urban and rural populations in five geographical regions corresponding to the first survey. Three provinces from each region, six counties from each province, and three urban districts and three rural villages from each county were randomly selected. Overall, the survey was carried out in 540 centers across the country.

### Sample size

Sample size for each region was determined by allowing for 1 % error in the expected prevalence of 10 %, and settled for both urban and rural areas, separately [12]. The number of people invited from each center was calculated on the basis of age distribution of the urban and rural populations in the relevant province [13].

People were selected from the local family health care center registries. In the Turkish health system, each family health care center serves 2,500–4,000 residents that include all residents in the area [11]. Every fifth person in the health registry was invited to participate. Participation was confirmed by telephone in the urban areas, and by house visits in the rural areas. The overall response rate was 87 %.

### Survey teams

Each team was comprised 2–3 members (a family physician, and/or a nurse, and a health technician, or a midwife). In total, 1,082 team members were involved in the field work. Seven to three days preceding the survey, team members attended a training course. A mobile core team was responsible for quality control, and logistics.

### Study protocol

Participants arrived at the survey center early in the morning after an overnight fast (≥10 h). The duration of fasting was also checked during participation via the questionnaire. We excluded those that reported <10 h of fasting from the analysis. A fasting venous blood sample was taken from all participants for plasma glucose (FPG), creatinine, lipid profile (total and HDL-cholesterol, and triglycerides), insulin, HbA_1c_, and hormones. At the same time, a fasting capillary blood glucose was also measured. People who had a FPG ≥ 7.0 mmol/L (≥126 mg/dL), and/or were on antidiabetic treatment were considered to have diabetes. The rest requested to drink 75 g anhydrous glucose dissolved in 250 mL water within 5 min, during the OGTT 1-, and 2-h capillary blood glucose levels (1- and 2-hPG) were measured.

A questionnaire was administered to collect data on social and demographic characteristics, medical history, lifestyle (e.g. education, socio-economic status [SES], physical activity, nutrition, alcohol and tobacco use) and reproductive history (women only). Systolic and diastolic blood pressures (sBP, dBP), heart rate, weight, height, waist and hip circumferences were measured according to the standard protocols [14, 15]. Body mass index (BMI) and waist-to-hip ratio (WHR) were calculated.

The study was approved by the local institutional ethical board (Istanbul University Faculty of Medicine Ethical Committee: 16.4.2008/699), therefore, all human studies have been performed in accordance with the ethical standards stated in the Declaration of Helsinki (Amendment-2004). And a written informed consent was provided by each participant.

### Laboratory tests

Capillary blood glucose concentrations were measured using a glucometer (Accu-CHEK Go; Roche Diagnostics, Germany), which uses a glucose oxidase method of estimation and gives values calibrated for plasma glucose. Performance of the device was compared with a glucose autoanalyzer (Roche System) and was found to be sufficiently reliable (n = 110; intra-assay CV, 1.8 %). During the field survey, instruments were checked every morning and whenever required with standard low and high glucose solutions.

All other biochemical tests were measured by the Roche Diagnostics Modular Autoanalyzer System [16] in the Central Biochemistry Laboratory of Istanbul Faculty of Medicine. HbA_1c_ levels were measured in whole blood samples by a turbidimetric inhibition immunoassay. Both the system and the laboratory have been regularly certified (Roche Diagnostics TQ HbA1c Gen. 3. NGSP Certificate of Traceability. September 2010–2011). CVs for reference normal, high, and intermediate HbA_1c_ were 2.9, 4.1, and 2.1 %, respectively (CAP GH2 Survey Data 5/2010).

As triglyceride levels might be affected by hyperglycemia, we used nonHDL-cholesterol instead of estimated LDL-cholesterol (nonHDL-cholesterol = total cholesterol minus HDL-cholesterol) [17]. Glomerular filtration rate (eGFR) was estimated by the ‘Modification of Diet in Renal Disease’ equation [18]. Insulin resistance was estimated using the following equation: ‘HOMA-IR = [Glucose (mmol/L) × Insulin (pmol/L)/22.5 × 6.945]’ [19]. A complete list of all laboratory analyses can be seen online in the Supplementary Table S1.

### Assessment of diabetes and prediabetes

Previously known diabetes was confined to self-reported cases under anti-diabetic treatment. Those who reported diabetes but not on anti-diabetic medications were confirmed with at least one of the three diagnostic tests (FPG, HbA_1c_ or OGTT). New diabetes and prediabetes (impaired fasting glucose [IFG], and IGT) were defined according to Expert Committee recommendations [20, 21]. FPG levels 5.6–6.9 mmol/L (100–125 mg/dL) but 2-hPG levels <7.8 mmol/L (<140 mg/dL) denoted ‘isolated-IFG’, 2-hPG levels 7.8–11.0 mmol/L (140–199 mg/dL) but FPG levels <5.6 mmol/L (<100 mg/dL) denoted ‘isolated-IGT’, and both FPG levels 5.6–6.9 mmol/L (100–125 mg/dL) and 2-hPG levels 7.8–11.0 mmol/L (140–199 mg/dL) denoted ‘combined prediabetes’ (IFG + IGT). Diagnosis of type 1 diabetes was beyond the scope of this survey.

### Definition of hypertension and obesity

Hypertension was defined as sBP ≥ 140 mmHg and/or dBP ≥ 90 mmHg or if a person was on regular antihypertensive treatment [22]. Obesity was defined as BMI ≥ 30 kg/m^2^ and overweight as 25–29.9 kg/m^2^. Central obesity was defined as waist ≥102 cm in men and ≥88 cm in women [15].

### Statistical methods

All analyses were performed using SPSS for Windows (version 19.0; SPSS/IBM, Chicago, IL). The χ^2^, the student’s t, and ANCOVA tests were used when appropriate. Pearson’s, or Spearman’s CVs, ORs, and 95 % CIs were calculated. A *p* value <0.05 was considered statistically significant. The prevalence of diabetes and prediabetes was estimated by 5-year age groups for both genders separately. Logarithmic transformations of nonhomogenously distributed factors were used. Variables that were associated with diabetes in the univariate analysis were included in the multiple logistic regression (backward elimination) models in men and women, separately.

To generate nationally and internationally-comparable results, the age-standardized prevalence was calculated using the ‘TURDEP-I’, ‘TurkStat-2009’, ‘WHO’s new World’ and ‘European’ populations as standards [6, 13, 23, 24].

## Results

In total, 26,499 people (63 % women) participated in the survey (urban: 15,783, rural: 10,441). The mean age of the participants was 45.8 years (SD, 15.3 years). Men were slightly older than women (Table 1).

**Table 1:** General features of the TURDEP-II population*

	Women (n = 16,696) Mean ± SD (95 % CI; range; interquartile range)	Men (n = 9,327) Mean ± SD (95 % CI; range; interquartile range)
Age (year)	44.6 ± 15.1 (44.4–44.9; 75.0; 23.0)	46.2 ± 15.8 (45.9–46.5; 70.0; 25.0)
Height (cm)	158.5 ± 6.8 (158.4–158.6; 66.0; 9.0)	171.2 ± 7.3 (171.1–171.4; 59.0; 10.0)
Weight (kg)	73.1 ± 14.5 (72.9–73.3; 121.0; 19.0)	80.3 ± 13.6 (80.0–80.6; 129.0; 18.0)
BMI (kg/m^2^)	29.2 ± 5.9 (29.1–29.3, 44.7; 8.0)	27.4 ± 4.4 (27.3–27.5; 47.1; 5.7)
Waist (cm)	92.8 ± 14.8 (92.5–93.0; 174.0; 20.0)	97.1 ± 13.0 (96.9–97.4; 150.0; 16.0)
Hip (cm)	109.6 ± 13.6 (109.4–109.8; 174.0; 16.0)	105.5 ± 10.5 (105.2–105.7; 155.0; 10.0)
WHR	0.846 ± 0.087 (0.845–0.847; 1.15; 0.11)	0.921 ± 0.087 (0.919–0.923; 1.57; 0.09)
sBP (mmHg)	120 ± 27 (119–120; 180; 30)	121 ± 23 (121–122; 170; 20)
dBP (mmHg)	74 ± 13 (74–75; 150; 12)	75 ± 12 (75–76; 130; 10)
Pulse (beat/min)	80 ± 9 (80–80; 44; 10)	78 ± 9 (78–79; 44; 12)
Smoking^a^		
Current user	9.8 (8.4–11.3)	31.4 (29.7–33.1)
Ex-smoker	5.2 (3.7–6.7)	25.1 (23.3–26.9)
Alcohol^a^		
Current user	1.5 (0.0–3.0)	17.5 (15.7–19.4)
Ex-user	0.5 (0.0–2.0)	5.9 (3.9–7.9)
Education^a^		
Illiterate	23.7 (22.4–27.7)	4.7 (2.7–5.8)
Literate, but no formal education	9.5 (8.1–11.8)	6.6 (4.7–7.3)
Education ≤5 years	45.6 (44.5–47.1)	43.8 (42.2–44.0)
Elementary school	7.1 (5.6–8.5)	13.6 (11.9–16.0)
High school	9.0 (7.5–10.3)	18.7 (15.6–19.3)
University	5.1 (2.4–5.3)	12.6 (10.3–14.1)

*sBP* systolic BP, *dBP* diastolic BP

* *p* < 0.001 for all variables between women and men

^a^Smoking, alcohol and education expressed as % (95 % CI)

The crude prevalence of diabetes was 16.5 % (95 % CI: 16.1–17.0); of them 45.5 % had newly diagnosed (prevalence: 7.5 %; 95 % CI: 6.3–8.7) and 54.5 % previously known (9.0 %; 7.8–10.1) diabetes (*p* < 0.001*).* Among previously known diabetes 85.5 % was on anti-diabetic medications (OAD: 71.9 %, insulin: 2.2 %, insulin + OAD: 11.4 %).

New diabetes was more common in the Eastern, Southern and Central regions; whereas known diabetes was more common in the Western and Northern regions (data not shown).

The crude prevalence of prediabetes was 30.8 % (isolated-IFG 14.7 %, isolated-IGT 7.9 %, and combined 8.2 %). Of the study population 36 % were obese and another 37 % overweight; central obesity was detected in 54 % and 31 % had hypertension (Supplementary Table S2).

The prevalence of age-standardized diabetes to several populations was as follow; the ‘TURDEP-I’: 13.7 %, ‘TurkStat-2009’ (official adult population in Turkey): 13.7 %, ‘WHO’s new World’: 15.0 %, and ‘European’: 17.1 %. The corresponding age-standardized prevalence for prediabetes, obesity, overweight, central obesity, and hypertension can be seen in Supplementary Table S2.

Diabetes was more common in women (17.2 %; 16.6–17.8) than men (16.0 %; 15.3–16.7) (*p* = 0.008), and in the urban (17.0 %; 16.4–17.6) than the rural (15.5 %; 14.8–16.2) population (*p* = 0.001).

**Fig. 1:**
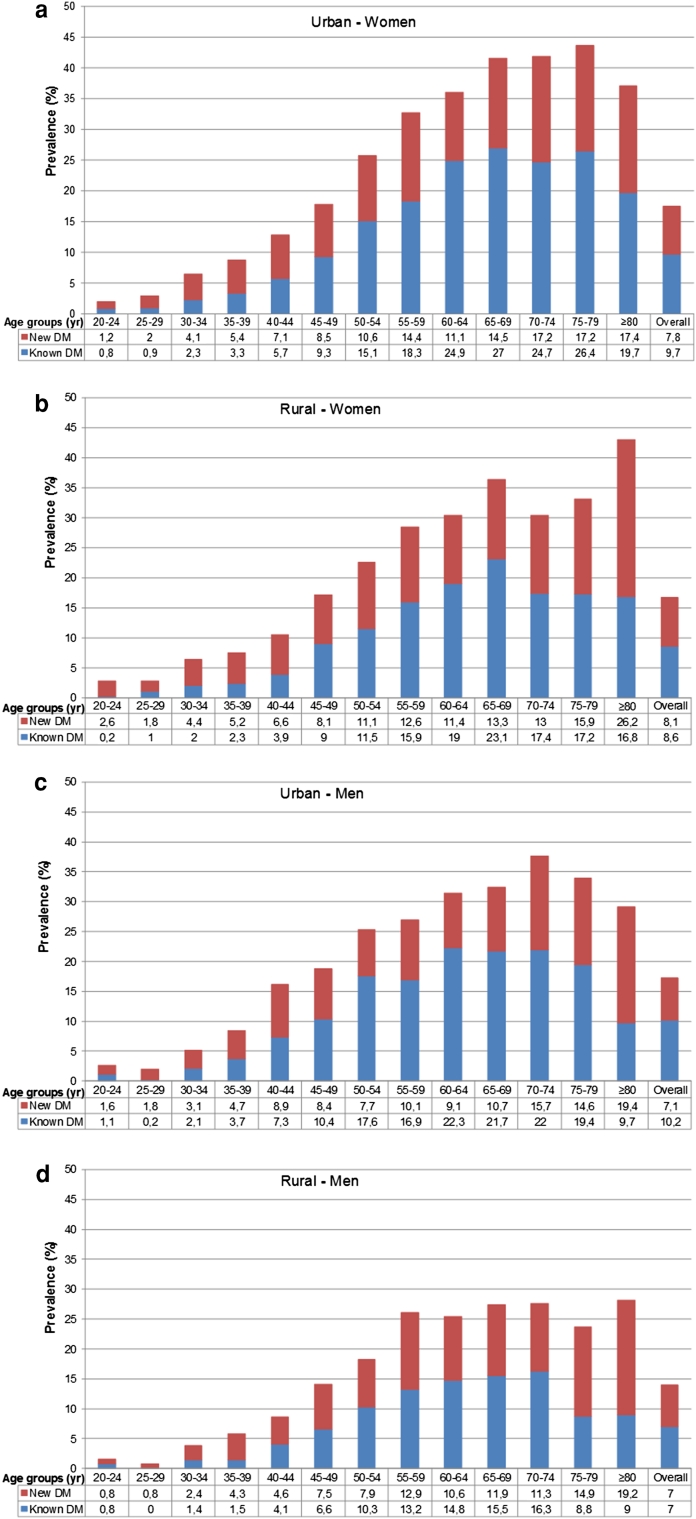
The prevalence of newly diagnosed and previously known diabetes by 5-year age intervals (**a** Urban - Women, **b** Rural - Women, **c** Urban - Men, and **d** Rural - Men)

**Fig. 2:**
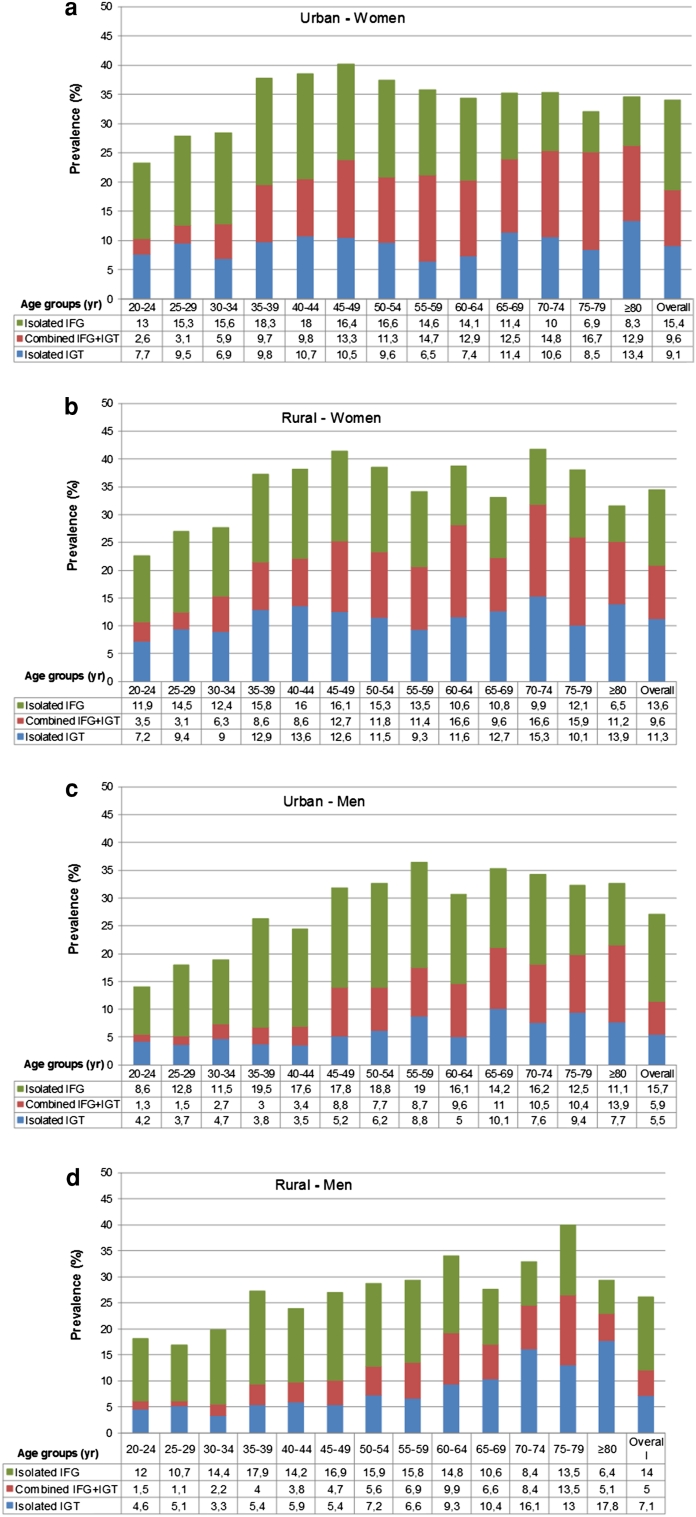
The prevalence of prediabetes (IFG, IGT, and combined) by 5-year age intervals (**a** Urban - Women, **b** Rural - Women, **c** Urban - Men, and **d** Rural - Men)

The prevalence of new and known diabetes in women was 7.8 and 9.7 % in urban, and 8.1 and 8.6 % in rural areas; the corresponding rates for men were 7.1 and 10.2 % in urban, and 7.0 and 7.0 % in rural (Fig. 1a–d). Prediabetes was also more common in women than men (*p* < 0.001). Isolated–IFG did not differ between genders; however, both isolated-IGT and combined prediabetes were more prevalent in women than in men (*p* < 0.001). Overall prediabetes did not differ between urban and rural. In contrast, both isolated-IFG (*p* = 0.001) and isolated-IGT (*p* < 0.001) were more common in urban than rural (data not shown). In the urban and rural areas, the overall prevalence of prediabetes in women was 34.1 and 34.5 %; and in men 27.1 and 26.1 %, respectively (Fig. 2a–d). Older (65–79 years in the urban, and ≥80 years in the rural) and younger (<45 years in both) participants had lower awareness of their diabetes.

The main characteristics of the survey population were evaluated according to the glucose tolerance status. There were a few gender differences in several characteristics as shown in Supplementary Table S3. Men in all categories except that for known diabetes were significantly older than women. In all categories, women had a significantly higher BMI, smaller waist and WHR (as expected) than men. In people with normal glucose regulation and in all prediabetes groups, women had a significantly lower sBP than men, whereas dBP was significantly higher in men only in the normal and isolated-IFG groups.

In all except the known diabetes group, significantly more women than men reported a family history of diabetes. Obesity and central obesity were more common among women than men, whereas being overweight was more prevalent among men. Hypertension was more prevalent among men with isolated-IGT and among women with known diabetes.

Women in the normal and isolated-IGT groups had remarkably higher FPG levels than men, while in the new diabetes group men had higher FPG levels than women. Mean 1-hPG level was higher in women than men in the normal, isolated-IGT and new diabetes groups; but mean 2-hPG level was significantly higher in women than men only in the normal and isolated-IFG groups. Mean HbA_1c_ was significantly higher in women than men in the normal group but lower in both new and known diabetes groups.

Mean serum creatinine level was markedly higher in men than women in all categories, but eGFR was significantly lower in men than women in the isolated-IGT group only.

Mean HOMA-IR was slightly higher in men than women in the new diabetes group. The mean high sensitive C-reactive protein level was markedly higher in women than men in the normal and known diabetes groups.

In all glucose categories, men had higher triglycerides and lower HDL-cholesterol levels than women. In the normal and isolated-IFG groups men had significantly higher non-HDL-cholesterol levels but only in the known diabetes group men had lower nonHDL-cholesterol levels than women.

Factors associated with the risk of new diabetes were evaluated by multiple logistic regression models (Table 2). In women, one SD (15.3 years) increase in age was associated with a 1.6 fold increase in the prevalence of diabetes. Similarly, each one SD increase in waist (14.8 cm) and BMI (5.9 kg/m^2^) was associated with a 1.16 and 1.09 times higher prevalence, respectively. Compared with the people in the North of Turkey, people living in all other regions had a 1.29–1.73 times higher risk of diabetes. Women who had not completed formal 8 years education had a 1.45 times higher risk than the more educated women. Having hypertension was associated with a 1.28–1.59 times increased risk. Each one person increase in the nuclear family was associated with a 1.04 times increased risk. Current cigarette smoking was the only factor associated with a decreased risk of diabetes by 26 % (OR: 0.74; 95 % CI: 0.54–0.98) in women.

**Table 2:** Factors associated with the risk of new diabetes mellitus in the population of TURDEP-II survey

Variable^a^	B	*p* value	OR	95 % CI
*WOMEN*				
Age (1 SD; 15.1 years)	0.473	<0.001	1.60	1.48–1.73
Region (North = 1)				
South	0.406	<0.001	1.50	1.22–1.84
West	0.256	0.019	1.29	1.04–1.59
East	0.553	<0.001	1.73	1.40–2.15
Central	0.341	0.002	1.40	1.13–1.73
Education (formal 8 years education = 1)				
<8 years	0.374	0.031	1.45	1.03–2.04
>8 years	–0.035	0.96	0.96	0.63–1.46
Family history of DM (no = 1)				
Positive family history	0.116	0.075	1.12	0.98–1.27
Hypertension (no = 1)				
Moderate	0.248	0.001	1.28	1.10–1.48
Severe	0.465	<0.001	1.59	1.23–2.06
Waist girth (1 SD; 14.8 cm)	0.151	<0.001	1.16	1.07–1.26
BMI (1 SD; 5.9 kg/m^2^)	0.089	<0.001	1.09	1.01–1.18
Smoking (no = 1)				
Current smokers	–0.301	0.036	0.74	0.54–0.98
Quitters	–0.289	0.089	0.74	0.53–1.04
Number of meal per day (≥5 = 1)				
3–4 meal per day	0.481	0.053	1.61	0.99–2.63
1–2 meal per day	0.379	0.135	1.46	0.88–2.40
Family size (1 person)	0.035	0.026	1.03	1.01–1.06
*MEN*				
Age (1 SD; 15.8 years)	0.514	<0.001	1.67	1.50–1.86
Hypertension (no = 1)				
Moderate	0.314	0.195	1.14	0.93–1.40
Severe	0.610	0.001	1.84	1.27–2.64
Social status (married = 1)				
Widow/separate	0.272	0.241	1.31	0.83–2.06
Single	–0.691	0.013	0.50	0.29–0.86
BMI (1 SD; 4.4 kg/m^2^)	0.254	<0.001	1.28	1.14–1.43
Family size (1 person)	0.043	0.06	1.04	0.99–1.09

*DM* diabetes mellitus

^a^Variable(s) entered on step 1: Age, social status, SES, education, waist, BMI, hypertension, income, alcohol, smoking, family history of DM, physical activity, region, urban/rural settlement, family size, and number of meal per day

Similarly, in men each one SD (15.8 years) increase in age and BMI (4.4 kg/m^2^) was associated with a 1.67 and 1.28 times increased risk of new diabetes, respectively. Hypertension was linked with a 1.84 times increased risk. The only factor linked with a reduced risk of new diabetes was being single compared with married men (OR: 0.50; 95 % CI: 0.29–0.86).

## Discussion

Compared with the data from the earlier TURDEP-I [6], the prevalence of diabetes, IGT, and obesity increased by 90, 106 and 40 %, respectively; but the prevalence of hypertension decreased by 11 %. The projected increases in the estimated numbers for diabetes, IGT, and obesity are largely, but not alone, attributable to the aging of the Turkish population, as the average life expectancy (from birth) between 2000 and 2009 increased from 67 to 72 years in men, and from 73 to 77 years in women [3]. Changing lifestyles in both urban and rural areas, and longer life survival of people with diabetes are other accountable factors for the increase in the prevalence of diabetes.

During the past 12 years the mean weight, height, waist, and hip measurements increased by 8 kg, 1 cm, 7 cm, and 3 cm in men; and by 6 kg, 1 cm, 6 cm, and 7 cm in women, respectively. The recent National Tobacco Control Program in Turkey successfully reduced the smoking rate particularly among men [25]. This may be contributed to some extent to the rapid increase in obesity and diabetes in men. In the present survey, we found that men but not women who had quitted smoking were significantly heavier and had a larger waist than those who had never smoked (*p* = 0.001, and *p* < 0.001, data not shown).

Some of the increase in the prevalence of diabetes in this survey may arise from a change in the diagnostic cut-off level of FPG between TURDEP-I and TURDEP-II. In TURDEP-I [6], we used the previous WHO criteria [10], i.e. individuals who self-reported diabetes and had fasting capillary blood glucose levels ≥7.8 mmol/L, and/or being under any glucose lowering treatment were considered to have diabetes, the rest had a OGTT. However, in the TURDEP-II, we applied current criteria [20, 21]. There were 364 people who self-reported diabetes but did not receive any anti-diabetic treatment and whose FPG level was 6.9–7.7 mmol/L; only 11 of them had 2-hPG levels >7.7 mmol/L, confirming that a recruitment bias due to self-reported diabetes was minimal in TURDEP-II. Therefore the present study is one of the very few nationwide surveys of diabetes and impaired glucose regulation truly based on currently recommended criteria and diagnostic classification worldwide.

However, if the same diagnostic definition at the time of TURDEP-I was applied in the current survey and the population’s age distribution was standardized to TURDEP-I population, the prevalence of diabetes should be 11.4 % (95 % CI: 11.0–11.8 % [men: 9.7; 95 % CI: 9.2–10.3 %, women: 12.7; 95 % CI: 12.1–13.3 %]). In this case diabetes should increase by 1.58 (men: 1.57, women: 1.59) times over 12 years, and the rate of increase should be calculated as 3.9 % per year.

The prevalence of OGTT-defined new diabetes was 4.9 % in TURDEP-II, compared with 2.3 % in TURDEP-I [6]; thus, it increased 2.13 times, i.e. on average 6.5 % per year over the last 12 years in Turkey. On the other hand, in this survey the prevalence of newly detected diabetes with FPG [21] and HbA_1c_ alone [26] was 4.2 and 3 %, respectively. Consequently, a OGTT, FPG or HbA_1c_ test alone could recognize only 65, 56 and 40 % of new diabetes cases according to the current criteria (data not shown). In our survey the mean levels of HbA_1c_ in the new diabetes group defined by FPG was 6.6 % (49 mmol/mol) and by OGTT was 5.9 % (41 mmol/mol). However, it was 7.6 % (60 mmol/mol) in the HbA_1c_-based new diabetes group. In several other population studies such as Chinese, Korean, Japan, Arab, Iranian, US and Australian it has been shown that HbA_1c_ as diagnostic method is less sensitive but more specific as compared to FPG or OGTT-based diagnosis [27–33]. Therefore, mean HbA_1c_ levels in people diagnosed by FPG is lower, and in those who diagnosed by OGTT is even lower than proposed cut-off levels.

While there is no doubt that the prevalence of diabetes has by now reached epidemic proportions in Turkey, it is interesting to compare the current results with the findings from other Turkish studies. In the TEKHARF study [4], carried out in 2004/2005, the prevalence of new diabetes in adults (≥35 years) was 11 %. The recently published CREDIT study [5] revealed that the diabetes prevalence (based on self-reported diabetes and FPG levels alone) was 12.7 % (women 14.3 % and men 10.9 %) in the population aged ≥ 18 years. A population-based survey of Turkish immigrants living in Sweden [34] indicated that the prevalence of diabetes based on a OGTT was 11.8 %.

Eastern Mediterranean and Middle East regions are considered to be on the verge of an emerging diabetes epidemic [1, 35, 36]. Some data exist in various countries in these regions to support this, but they are not based on nationwide samples and methods used in these studies do not comply with the current criteria and diagnostic classification of diabetes. The prevalence of diabetes in this study was similar to that in Qatar [37] (16.1 %), Syria [38] (15.6 %), and Oman [39] (16.7 %); but lower than in Bahrain [40] (25.7 %), Saudi Arabia [41] (23.7 %), and United Arab Emirates [42] (17.1 %). It was, however, higher than in European Mediterranean countries such as Cyprus (North [43]: 11.3 %, South [44]: 10.3 %) and Spain [45] (13.2 %). The results from our and the above mentioned populations confirmed a higher prevalence of diabetes in fast evolving countries than in the developed countries.

In this survey women had a higher diabetes prevalence than men that is considered unusual for many populations (i.e. Switzerland [46]: 9.1 % in men and 3.8 % in women; Japan [47]: 15.3 % in men and 7.3 % in women; Finland, middle-aged adults [48]: 10.2 % in men and 7.4 % in women, and also in the Collaborative European study [49]). This may be explained by a higher prevalence of obesity among middle-aged, and older Turkish women compared with men. A low level of physical activity due to traditional and cultural attitudes may also contribute to a higher rate of obesity and diabetes in women than men in Turkey.

Between TURDEP-I and TURDEP-II surveys, average age-standardized BMI increased from 26.6 to 28.6 kg/m^2^ and average waist increased from 87.2 to 94.5 cm over 12 years in Turkey. We calculated the difference in BMI and waist between the two surveys by 5-year age groups and analyzed across the increase in prevalence of diabetes over 12 years. The change in prevalence of diabetes is correlated with the change in BMI (r = 0.709, *p* = 0.015), and waist (r = 0.651, *p* = 0.030). In the current survey we have shown that in women, each one SD increase in waist (14.8 cm) and BMI (5.9 kg/m^2^) was associated with a 1.16 and 1.09 times higher prevalence of newly diagnosed diabetes. Similarly, in men each one SD (4.4 kg/m^2^) increase in BMI was associated with a 1.28 times increased risk of new diabetes (Table 2). BMI and WHR were also reported as factors associated with previously unknown diabetes in our first survey (6). Our results confirmed that obesity is one of the major contributing factors of diabetes epidemic.

With the recent improvements in SES, disparities have reduced differences between urban and rural areas in Turkey. The urban–rural difference in the prevalence of diabetes compared with TURDEP-I has been changed for new diabetes from 0.4 to −0.1 % and for known diabetes from 1.9 to 1.6 % (data not shown).

Compared with the TURDEP-I survey [6], diabetes awareness in the population has reduced, similar to many other populations [1, 36, 49]. Now, the ratio of new-to-known diabetes has increased from 1/2 in TURDEP-I to nearly 1/1 in TURDEP-II. This observation may also reconfirm that there was no over diagnosis of diabetes in this survey.

In keeping with previous studies in Turkey and in other populations [4–6, 50–53] we found a significant inverse relationship between educational level and the prevalence of diabetes especially among women. This finding supports ongoing campaigns to increase girls’ enrolment to schools, since these are also associated with health benefits.

The only improved parameter from TURDEP-I to TURDEP-II was a 11 % decrease in the age-standardized prevalence of hypertension. We may explain this with reduced rate of smoking, and strong legislative regulations on salt-restriction in Turkey, i.e. the salt content of bread and all processed foods is reduced; salt-content of meals in all school, work-place and public cafeterias and restaurants are subject to reduce the amount of salt; in all restaurants salt is provided on request.

The strengths of this study are that it’s nationally representative design, large size and a high response rate. In addition, nationwide changes in prevalence of diabetes over 12 years period were demonstrated; such data hardly exist in any other country. We included various regions, and both urban and rural areas. For instance in the recent Chinese prevalence study, rural areas were located nearby large cities, and therefore may not provide the real picture of urban–rural difference in diabetes prevalence [54]. Further, we have collected data on the vital determinants of diabetes, i.e. anthropometrics, dietary intakes, physical activity, living environment, women’s reproductive data, and co-morbid conditions, along with a large number of biochemical tests. Therefore, we are able to evaluate the association between these factors and diabetes. Third, to ensure comparability across studies, we applied the OGTT and used the WHO criteria to define diabetes and prediabetes in our study. In addition, we determined HbA_1c_ in all survey participants. Thus, this survey was more comprehensive than other surveys recently carried out in other countries.

Limitations of the study include that, women and elderly people were slightly over-represented, although we took care of this issue by age standardization of the survey results to the 2009 official Turkish population published in 2010 by TurkStat [13].

Recent estimates of diabetes and predictions for the year 2030 calculated by WHO [36], and International Diabetes Federation [1] for different countries were based on available published papers. Nevertheless, individual data from several populations [37–42, 54] including ours have pointed out that those 2030 expectations have already been exceeded. The new ‘WHO Global Noncommunicable Disease Surveillance Report’ recommends that the member states should monitor the prevalence of diabetes [55]. The present study along with others has demonstrated that, without proper diabetes surveys, the magnitude of this major public health problem cannot be identified, and the trends cannot be determined.

TURDEP-II has provided a comprehensive and up-to-date review of the epidemiological trends and public health implications of diabetes in Turkey. The survey indicates that the prevalence of diabetes has drastically increased during the recent years, and now reached epidemic levels. We estimate that 6.5 million people in Turkey have diabetes, and almost a half of them are unaware of it. Another 14.5 million people have prediabetes, either IFG or IGT. These results are distressing and underscore the urgent need for the development of national strategies aiming to prevent diabetes and -in those already affected- to manage the illness effectively in order to prevent its complications. Moreover, this survey provides an example that systematic monitoring of the prevalence of diabetes and its risk factors at the population (and national) level is feasible, even in such a large country as Turkey. Such a fast rate of increase in diabetes prevalence found in this survey provided valuable data not only for local health authorities but also globally.

### Electronic supplementary material

Below is the link to the electronic supplementary material.

Supplementary material 1 (DOC 140 kb)

## Appendix: Members of The TURDEP-II Study Group

*Investigators*—I Satman, N Dinccag, K Karsidag, T Yilmaz, F Alagol, B Omer, S Kalaca, Y Tutuncu, NC Ozbey, H Boztepe, S Genc, S Gedik, F Turker, A Telci, B Canbaz, RS Calis, YM Oltulu; *The* *Ministry of Health*–B Cakir. B Keskinkilic, R Imamecioglu, N Yardim, N Coban; *Adviser* **—**J Tuomilehto; *Field survey*–AI Dokucu, D Ozkul, H Karabulut, I Topcu, SB Kartal, S Cinar, A Uzunoglu, T Kirtas, E Ucuncuoglu, O Altinkaynak, C Kahveci (Istanbul); YC Sahin, E Saydam, D Gurgenyatagi, G Hamzaoglu, M Demirci (Samsun); M Dereli (Trabzon); A Akkaya, Y Bas, G Ozdemir, YC Guneyler, M Derin (Bursa); AO Candan (Izmir); M Okudan (Antalya); NN Colak (Adana); M Akoz (Gaziantep); M Gundogdu (Denizli); E Gurgut (Erzurum); G Kuzu, HB Zengin, D Bozkurt (Malatya); D Bilici, M Zafer (Diyarbakir); M Erogul, A Sag, A Simsek, A Altin, U Cakar (Eskisehir); T Ozdemir, Y Gokce (Ankara); A Sakir, O Unsal, N Uyar, S Akdeniz (Konya); *Universities & Training*-*Research Hospitals*–S Akalin, E Ozer, Y Altuntas, M Sargin, A Sengul, S Salman, F Salman, A Turkmen (Istanbul); S Imamoglu, OO Gul (Bursa); C Yilmaz, F Saygili, S Cetinkalp, F Bayraktar, S Yesil, A Comlekci, M Bahceci, GG Oruk (Izmir); M Balci. H Altunbas, BU Koyuncu (Antalya); T Tetiker (Adana); M Araz, E Akarsu (Gaziantep); A Tuzcu (Diyarbakir); I Sahin, AC Sertkaya (Malatya); G Akcay (Erzurum); A Kaya, S Gonen (Konya); M Arslan, S Gullu, G Ayvaz, A Corakci, M Kutlu, T Erbas, M Bayraktar, N Baskal, B Cakir, S Guler (Ankara); B Efe, A Akalin, G Yorulmaz (Eskisehir); F Akin. E Yerlikaya (Denizli); A Atmaca, EK Kan (Samsun); C Erem, HO Ersoz, I Nuhoglu, E Algun (Trabzon); *Monitor CRO*—S Misirlioglu, G Betin, E Koruyucu, A Calisgan, O Akbas, T Devlen, G Okyay, E Erdem, C Sarp, F Durgun, C Akbas, S Fesligil, M Sasmaz; *Other supporters*–O Halil, H Kirmaz, H Oget, C Sengor, B Sakkaoglu, M Tanberk, M Satman, A Koroglu, Y Ersahin, S Uygur, A Aydin.

## References

[1] International Diabetes Federation (2011). Diabetes atlas.

[2] World Health Organization Europe (2009). The European health report 2009: health and health systems.

[3] World Health Organization (2011). World health statistics 2011.

[4] Onat A, Hergenc G, Uyarel H, Can G, Ozhan H (2006). Prevalence, incidence, predictors and outcome of type 2 diabetes in Turkey (Turkiye’de tip 2 diyabetin prevalansi, insidansi, ongorduruculeri ve akibeti). Anadolu Kardiyol Derg.

[5] Suleymanlar G, Utas C, Arinsoy T, Arınsoy T, Ates K, Ecder T, Camsari T, Serdengecti K (2011). A population-based survey of Chronic REnal disease in Turkey-the CREDIT study. Nephrol Dial Transpl.

[6] Satman I, Yilmaz T, Sengul A, Salman S, Salman F, Uygur S, Bastar I, Tutuncu Y, Sargın M, Dinccag N, Karsidag K, Kalaca S, Ozcan C, King H, The TURDEP Group. Population-based study of diabetes and risk characteristics in Turkey: results of the Turkish diabetes epidemiology study (TURDEP). Diabetes Care. 2002;25:1551–6. 10.2337/diacare.25.9.1551 12196426

[7] Bagriacik N, Ipbuker A, Ilkova H. Diabetes in Turkey. IDF Bull. 1990;XXXV:3.

[8] Kelestimur F, Cetin M, Pasaoglu H, Coksevim B, Cetinkaya F, Unluhizarci K (1999). The prevalence and identification of risk factors for type 2 diabetes mellitus and impaired glucose tolerance in Kayseri, Central Anatolia, Turkey. Acta Diabetol.

[9] King H, Minjoot-Pereira G (1999). Diabetes and noncommunicable disease risk factor surveys: a field guide.

[10] World Health Organization. Diabetes Mellitus. Report of a WHO Study Group. Technical report series no. 727. Geneva: WHO Publication; 1985. 3934850

[11] Ministry of Health, Hacettepe University Institute for Population Studies, Macro International Inc. Turkey Population Health Survey 1993. Ankara: Hacettepe University; 1994.

[12] Lwanga SK, Lemeshow S (1991). Sample size determination in health studies: a practical manual.

[13] The Results of the Address-Based Population Registration System in 2008–2011. In: Turkish Statistical Institute (TurkStat). http://www.tuikapp.tuik.gov.tr/adnksdagitapp/adnks.zul. Accessed 28 Feb 2011.

[14] Dowse GK, Zimmet P (1992). A model protocol for diabetes and other noncommunicable disease survey. World Health Stat Q.

[15] World Health Organization (1997). Report of a WHO consultation on obesity.

[16] ELECSYS, COBAS, COBAS E and LIFE NEEDS ANSWERS. In: Biochemical tests reference intervals for children and adults, 2008. Roche Diagnostics, Germany. http://www.roche.com. Accessed 1 Sept 2008.

[17] Lu W, Resnick HE, Jablonski KA, Jones KL, Jain AK, Howard WJ, Robbins DC, Howard BV (2003). Non-HDL cholesterol as a predictor of cardiovascular disease in type 2 diabetes: The Strong Heart Study. Diabetes Care.

[18] Rigalleau V, Lasseur C, Perlemoine C, Barthe N, Raffaitin C, Liu C, Chauveau P, Baillet-Blanco L, Beauvieux MC, Combe C, Gin H (2005). Estimation of glomerular filtration rate in diabetic subjects: Cockcroft formula or Modification of Diet in Renal Disease study equation?. Diabetes Care.

[19] Matthews DR, Hosker JP, Rudenski AS, Naylor BA, Treacher DF, Turner RC (1985). Homeostasis model assessment: insulin resistance and beta-cell function from fasting plasma glucose and insulin concentrations in man. Diabetologia.

[20] The Expert Committee on the Diagnosis and Classification of Diabetes Mellitus. Report of the Expert Committee on the diagnosis and classification of diabetes mellitus. Diabetes Care. 1998;21(Suppl. 1):S5–19.

[21] The Expert Committee on the Diagnosis and Classification of Diabetes Mellitus (2003). Follow-up report on the diagnosis of diabetes mellitus. Diabetes Care.

[22] Ahmad BO, Boschi-Pinto C, Lozano R, Inoue M. Age standardization of rates: a new WHO standard. GPE discussion paper series: no. 31, Geneva: EIP/GPE/EBD WHO; 2001. p. 1–14.

[23] Chobanian AV, Bakris GL, Black HR, Cushman WC, Green LA, Izzo, JL Jr, Jones DW, Materson BJ, Oparil S, Wright JT Jr, Roccella EJ, The National High Blood Pressure Education Program Coordinating Committee. 2003. The National High Blood Pressure Education Program Coordinating Committee, JNC 7: complete report. Seventh Report of the Joint National Committee on Prevention, Detection, Evaluation, and Treatment of High Blood Pressure. Hypertension. 2003;42:1206–52. 10.1161/01.HYP.0000107251.49515.c2 14656957

[24] West Midlands Public Health Observatory. In: Metadata: The European Standard Population, 2007. http://www.wmpho.org.uk/localprofiles/metadata.aspx?id=META_EUROSTD. Accessed 28 Feb 2011.

[25] The Ministry of Health of Turkey (2010). Global Adult Tobacco Survey—Turkey report.

[26] The International Expert Committee (2009). International Expert Committee Report on the role of the A1C assay in the diagnosis of diabetes. Diabetes Care.

[27] Zhou X, Pang Z, Gao W, Wang S, Zhang L, Ning F, Qiao Q (2010). Performance of an A1C and fasting capillary blood glucose test for screening newly diagnosed diabetes and pre-diabetes defined by an oral glucose tolerance test in Qingdao, China. Diabetes Care.

[28] Choi SH, Kim TH, Lim S, Park KS, Jang HC, Cho NH (2011). Hemoglobin A1c as a diagnostic tool for diabetes screening and new-onset diabetes prediction. Diabetes Care.

[29] Takahashi Y, Noda M, Tsugane S, Kuzuya T, Ito C, Kadowaki T (2000). Prevalence of diabetes estimated by plasma glucose criteria combined with standardized measurement of HbA_1c_ among health checkup participants on Myako Island, Japan. Diabetes Care.

[30] Pinelli NR, Jantz AS, Martin ET, Jaber LA (2011). Sensitivity and specificity of glycated hemoglobin as a diagnostic test for diabetes and prediabetes in Arabs. JCEM.

[31] Ghazanfari Z, Haqhdoost AA, Alizadeh SM, Atapour J, Zolala F (2010). A comparison of HbA1c and fasting blood tests in general population. Int J Prev Med.

[32] Rohlfing CL, Little RR, Wiedmeyer HM, England JD, Madsen R, Harris MI, Flegal KM, Eberhardt MS, Goldstein DE (2000). Use of GHb (HbA1c) in screening for undiagnosed diabetes in the U.S. population. Diabetes Care.

[33] Colagiuri S, Hussain Z, Zimmet P, Cameron A, Shaw J, AusDiab. Screening for type 2 diabetes and impaired glucose metabolism: the Australian experience. Diabetes Care. 2004;27:367–71. 10.2337/diacare.27.2.367 14747215

[34] Hjörleifsdottir-Steiner K, Satman I, Sundquist J, Kaya A, Wändell P (2011). Diabetes and impaired glucose tolerance among Turkish immigrants in Sweden. Diabetes Res Clin Pract.

[35] King H, Aubert RE, Herman WH (1998). Global burden of diabetes, 1995–2025. Diabetes Care.

[36] Wild S, Roglic G, Green A, Sicree R, King H (2004). Global prevalence of diabetes: estimates for the year 2000 and projections for 2030. Diabetes Care.

[37] Bener A, Zirie M, Janahi IM, Al-Hamaq AOAA, Musallam M, Wareham NJ (2009). Prevalence of diagnosed and undiagnosed diabetes mellitus and its risk factors in a population-based study of Qatar. Diabetes Res Clin Pract.

[38] Albache N, Al Ali R, Rastam S, Fouad FM, Mzayek F, Maizak W. Epidemiology of type 2 diabetes in Aleppo, Syria. J Diabetes. 2010;2:85–91. 10.1111/j.1753-0407.2009.00063.x 20923489

[39] Al-Lawati JA, Al Riyami AM, Mohammed AJ, Jousilahti P. Increasing prevalence of DM in Oman. Diabet Med. 2002;19:954–7. 10.1046/j.1464-5491.2002.00818.x 12421434

[40] Hamadeh RR (2000). Non-communicable diseases among the Bahraini population: a review. East Mediterr Health J.

[41] Al-Nozha MM, Al-Maatouq MA, Al-Mazrou YY, Arafah MR, Khalil MZ, Khan NB, Al-Marzouki K, Abdullah MA, Al-Khadra AH, Al-Harthi SS, Al-Shahid MS, Al-Mobeireek A, Nouh MS (2004). Diabetes mellitus in Saudi Arabia. Saudi Med J.

[42] Saadi H, Carruthers SG, Nagelkerke N, Al-Maskari F, Afandi B, Reed R, Lukic M, Nicholls MG, Kazam E, Algawi K, Al-Kaabi J, Leduc C, Sabri S, El-Sadig M, Elkhumaidi S, Agarwal M, Benedict S (2007). Prevalence of diabetes mellitus and its complications in a population-based sample in Al Ain, UAE. Diabetes Res Clin Pract.

[43] Satman I, Yilmaz MT, Karsidag K, Dinccag N, Sengul A, Salman S, Yillar G, Salman F, Tasyurek A, Sav H, Karadeniz S, Sargin M. Northern Cyprus: another high prevalence area of diabetes and impaired glucose tolerance in the Mediterranean (Abstract). Diabetologia. 1997;40(Suppl. 1):A185.

[44] Loizou T, Pouloukas S, Tountas C, Thanopoulou A, Karamanos V (2006). An epidemiologic study on the prevalence of diabetes, glucose intolerance, and metabolic syndrome in the adult population of the republic of Cyprus. Diabetes Care.

[45] Boronat M, Varillas VF, Saavedra P, Suárez V, Bosch E, Carrillo A, Nóvoa FJ (2006). DM and impaired glucose regulation in the Canary islands (Spain) prevalence and associated factors in the adult population of Telde, Gran Canaria. Diabet Med.

[46] Kaiser A, Vollenweider P, Waeber G, Marques-Vidal P (2012). Prevalence, awareness and treatment of type 2 diabetes mellitus in Switzerland: the CoLaus study. Diabet Med.

[47] Morimoto A, Nishimura R, Tajima N (2010). Trends in the epidemiology of patients with diabetes in Japan. Jpn Med Assoc J.

[48] Yliharsila H, Lindstrom J, Eriksson JG, Jousilahti P, Valle TT, Sundvall J, Tuomilehto J (2005). Prevalence of diabetes and impaired glucose regulation in 45- to 64-year-old individuals in three areas of Finland. Diabet Med.

[49] The DECODE Study (Diabetes Epidemiology: Collaborative Analysis of Diagnostic Criteria in Europe) Group. Consequences of the new diagnostic criteria for diabetes in older men and women. Diabetes Care. 1999;22:1667–71. 10.2337/diacare.22.10.1667 10526732

[50] Borrell LN, Dallo FJ, White K (2006). Education and diabetes in a racially and ethnically diverse population. Am J Public Health.

[51] Ko GT, Chan JC, Yeung VT, Chow CC, Tsang LW, Cockram CS (2001). A low socioeconomic status is an additional risk factor for glucose intolerance in high risk Hong Kong Chinese. Eur J Epidemiol.

[52] Seeman T, Merkin SS, Crimmins E, Koretz B, Charette S, Karlamangla A (2008). Education, income and ethnic differences in cumulative biological risk profiles in a national sample of US adults: NHANES III (1988–1994). Soc Sci Med.

[53] Yan LL, Liu K, Daviglus ML, Colangelo LA, Kiefe CI, Sidney S, Matthews KA, Greenland P (2006). Education, 15-year risk factor progression, and coronary artery calcium in young adulthood and early middle age: the Coronary Artery Risk Development in Young Adults study. JAMA.

[54] Yang W, Lu J, Weng J, Jia W, Ji L, Xiao J, Shan Z, Liu J, Tian H, Ji Q, Zhu D, Ge J, Lin L, Chen L, Guo X, Zhao Z, Li Q, Zhou Z, Shan G, He J (2010). China National Diabetes and Metabolic Disorders Study Group. Prevalence of diabetes among men and women in China. N Engl J Med.

[55] World Health Organization (2008). 2008–2013 Action plan for the global strategy for the prevention and control of noncommunicable diseases.

