# Supplementary material for: Twelve-year trends in the prevalence and risk factors of diabetes and prediabetes in Turkish adults
Source: Eur J Epidemiol. 2013 Feb 14;28(2):169–80. doi: 10.1007/s10654-013-9771-5 (PMC3604592; doi:10.1007/s10654-013-9771-5)
Supplement: Supplementary file 1 — Supplementary material 1 (DOC 140 kb) [file 10654_2013_9771_MOESM1_ESM.doc]

**Online supplementary tables**

| **Table S1** Laboratory parameters in TURDEP-II survey | | |
| --- | --- | --- |
| **Measured parameter** | **Measured parameter** | **Calculated parameters** |
| 1-hPG | hsCRP | eAG |
| 2-hPG | IGF1 | eGFR-CKD Epi |
| 25(OH)D* | IGFBP3 | eGFR-Cockroft |
| Anti-Tg | Insulin | eGFR-cystatin C |
| Creatinine | PTH* | eGFR-MDRD |
| Cystatin C | Total cholesterol | FT4/TSH |
| Folate* | TPOAbs | HOMA-IR |
| FPG | Triglycerides | LDL-cholesterol |
| FT4 | TSH | NonHDL-cholesterol |
| HbA_1c_ | Vitamin B12* |  |
| HDL-cholesterol | 25(OH)D* |  |
| 1-hPG, 1-h plasma glucose; 2-hPG, 2-h plasma glucose; 25(OH)D, 25 hydroxyvitamin D; Anti-Tg, anti-thyroglobulin antibodies; CKD Epi, chronic kidney disease epidemiology; eAG, estimated average glucose; eGFR, estimated GFR; FPG, fasting plasma glucose; FT4, free-T_4_; HbA_1c_, glycated hemoglobin A_1c_; HOMA-IR, Homeostasis model assessment-insulin resistanc; HDL-cholesterol, high density lipoprotein-cholesterol; ; hsCRP, high sensitive C-reactive protein; IGFBP3, IGF binding protein-3; LDL-cholesterol, low density lipoprotein-cholesterol; MDRD, Malnutrition Diet Renal Disease; PTH, parathyroid hormone; TPOAbs, thyroid peroxidase antibodies; TSH, thyroid stimulating hormone.  *Measured in a subgroup of the study population. | | |

| **Table S2** Crude and standardized prevalence of diabetes, prediabetes, obesity, overweight, central obesity, and hypertension in TURDEP-II population | | | | | |
| --- | --- | --- | --- | --- | --- |
| **Parameter** | **TURDEP-II**  **(2010)**  **Crude prevalence**  **(% [95% CI])** | **Standardized prevalence with standard population of:**  **(% [95% CI])** | | | |
|  |  | **TURDEP-I**  **(1997-98)** | **TurkStat-2009** | **World population** | **European population** |
| **Total diabetes (new + known)** | 16.5 (16.1-17.0) | 13.7 (13.2-14.1) | 13.7 (13.69-13.71) | 15.0 (14.5-15.4) | 17.1 (16.5-17.6) |
| **Isolated IFG** | 14.7 (14.4-15.3) | 14.5 (14.0-14.9) | 14.3 (14.29-14.31) | 14.2 (13.7-14.6) | 14.2 (13.7-14.6) |
| **Isolated IGT** | 7.9 (7.6-8.3) | 7.1 (6.7-7.4) | 7.6 (7.59-7.61) | 7.7 (7.4-8.0) | 7.9 (7.5-8.2) |
| **Combined IFG+IGT** | 8.2 (7.8-8.5) | 6.7 (6.4-7.0) | 7.2 (7.19-7.21) | 7.5 (7.2-7.8) | 7.9 (7.5-8.3) |
| **General obesity (BMI ≥30.0 kg/m^2^)** | 36.0 (35.3-36.5) | 31.2 (30.5-31.9) | 32.1 (32.09-32.11) | 33.0 (32.3-33.6) | 35.2 (34.5-35.9) |
| **Overweight (BMI 25.0-29.9 kg/m^2^)** | 37.0 (36.1-37.2) | 37.5 (36.7-38.2) | 36.0 (35.99-36.01) | 36.1 (35.7-36.6) | 36.5 (35.7-37.2) |
| **Central obesity (waist ≥88 cm for women; ≥102cm for men)** | 53.6 (52.8-54.1) | 46.3 (45.4-47.1) | 48.9 (48.89-48.91) | 50.2 (49.3-51.0) | 53.2 (52.3-54.0) |
| **Hypertension (sBP ≥140 mmHg and/or dBP ≥90 mmHg or on antihypertensive drug therapy)** | 31.4 (30.9-32.1) | 25.6 (24.9-26.2) | 26.5 (26.49-26.51) | 28.3 (27.6-28.9) | 32.0 (31.3-32.7) |
| IFG, impaired fasting glucose, IGT, impaired glucose tolerance. | | | | | |

| **Table S3** Age-adjusted characteristics of the survey population across to glucose tolerance categories | | | | | | | |
| --- | --- | --- | --- | --- | --- | --- | --- |
| **Parameter*** | **Normal GT**  (8020 F / 5269 M) | **Isolated IFG**  (2448 F / 1401 M) | **Isolated IGT**  (1540 F / 543 M) | **Combined ‘IFG+IGT’**  (1601 F / 518 M) | **New DM**  (1325 F / 658 M) | **Known DM**  (1556 F / 832 M) | **p value** (ANCOVA between categories) |
| **BMI (kg/m^2^)** | | | | | | | |
| **Women** | 27.8±5.7 | 29.4±5.7 | 28.9±5.4 | 31.3±5.7 | 31.0±6.1 | 32.1±5.7 |  |
| **Men** | 26.7±4.3  p<0.001 | 27.8±4.2  p<0.001 | 27.6±4.3  p<0.001 | 28.7±4.5  p<0.001 | 28.8±4.7  p<0.001 | 29.4±4.4  p<0.001 | p=0.026 |
| **Waist (cm)** | | | | | | | |
| **Women** | 89.5±14.7 | 93.0±14.0 | 91.9±13.8 | 97.0±13.5 | 98.8±14.5 | 101.9±12.8 |  |
| **Men** | 95.0±13.2  p<0.001 | 98.4±11.4  p<0.001 | 97.0±11.3  p<0.001 | 101.2±12.0  p<0.001 | 101.6±13.4  p<0.001 | 103.7±12.1  p=0.002 | p=0.009 |
| **WHR** | | | | | | | |
| **Women** | 0.83±0.09 | 0.84±0.08 | 0.85±0.09 | 0.86±0.08 | 0.88±0.09 | 0.89±0.08 |  |
| **Men** | 0.91±0.09  p<0.001 | 0.92±0.08  p<0.001 | 0.92±0.08  p<0.001 | 0.94±0.09  p<0.001 | 0.95±0.09  p<0.001 | 0.96±0.08  p<0.001 | p=0.001 |
| **sBP (mmHg)** | | | | | | |  |
| **Women** | 115±30 | 119±21 | 120±22 | 125±22 | 129±24 | 133±24 |  |
| **Men** | 117±19  p<0.001 | 121±30  p=0.010 | 125±23  p<0.001 | 129±22  p=0.006 | 130±23  p=0.527 | 131±23  p=0.130 | p=0.001 |
| **dBP (mmHg)** | | | | | | | |
| **Women** | 72±13 | 75±13 | 75±13 | 78±13 | 79±15 | 80±13 |  |
| **Men** | 74±12  p<0.001 | 75±12  p=0.193 | 77±14  p<0.001 | 79±13  p=0.173 | 79±13  p=0.765 | 79±13  p=0.109 | p=0.042 |
| **Family history of diabetes (%)** | | | | | | | |
| **Women** | 40.8 (39.7-41.9) | 43.3 (41.3-43.3) | 40.3 (37.9-42.8) | 45.3 (42.9-47.7) | 42.3 (39.6-45.0) | 61.9 (59.5-64.3) |  |
| **Men** | 33.5 (32.3-34.8)  p<0.001 | 38.5 (36.0-41.1)  p=0.004 | 28.9 (25.1-32.7)  p<0.001 | 33.8 (29.7-37.9)  p<0.001 | 35.1 (31.5-38.8)  p=0.002 | 59.0 (55.7-62.3)  p=0.387 | p<0.001 |
| **Overweight (%)** | | | | | | | |
| **Women** | 32.8 (31.8-33.8) | 32.4 (30.6-34.3) | 32.7 (30.4-35.0) | 29.8 (27.6-32.0) | 28.1 (25.7-30.5) | 27.6 (25.4-29.8) |  |
| **Men** | 43.8 (42.5-45.1)  p<0.001 | 46.8 (44.2-49.4)  p<0.001 | 44.8 (40.6-49.0)  p<0.001 | 48.5 (44.2-52.8)  p<0.001 | 38.3 (34.6-42.0)  p<0.001 | 42.8 (39.4-46.2)  p<0.001 | p=0.124 |
| **Obesity (%)** | | | | | | | |
| **Women** | 32.4 (31.4-33.4) | 41.8 (39.9-43.8) | 39.4 (37.0-41.8) | 54.8 (52.4-57.2) | 53.4 (50.7-56.1) | 58.6 (56.2-61.1) |  |
| **Men** | 20.4 (19.3-21.5)  p<0.001 | 26.9 (24.6-29.2)  p<0.001 | 25.0 (21.4-28.6)  p<0.001 | 32.8 (28.8-36.8)  p<0.001 | 38.0 (34.3-41.7)  p<0.001 | 39.8 (36.5-43.1)  p<0.001 | p=0.008 |
| **Central obesity (%)** | | | | | | | |
| **Women** | 53.4 (52.3-54.5) | 64.1 (62.2-66.0) | 61.6 (59.2-64.0) | 75.9 (73.8-78.0) | 77.2 (74.9-79.5) | 85.9 (84.2-87.6) |  |
| **Men** | 27.6 (26.4-28.8)  p<0.001 | 35.6 (33.1-38.1)  p<0.001 | 32.0 (28.1-35.9)  p<0.001 | 46.5 (42.2-50.8)  p<0.001 | 48.2 (44.4-52.0)  p<0.001 | 54.1 (50.7-57.5)  p<0.001 | p=0.004 |
| **Hypertension (%)** | | | | | | | |
| **Women** | 21.4 (20.5-22.3) | 30.5 (27.1-33.9) | 32.5 (30.2-34.8) | 45.0 (42.6-47.4) | 50.4 (47.7-53.1) | 67.8 (65.5-70.1) |  |
| **Men** | 22.5 (21.4-23.6)  p=0.116 | 28.3 (25.9-30.7)  p=0.143 | 40.3 (36.2-44.4)  p=0.001 | 49.0 (44.7-53.3)  p=0.116 | 47.4 (43.6-51.2)  p=0.215 | 57.7 (54.3-61.1)  p=0.001 | p=0.002 |
| **FPG (mmol/L)** | | | | | | | |
| **Women** | 5.13±0.46 | 5.89±0.30 | 5.11±0.33 | 5.99±0.35 | 7.47±2.48 | 9.20±3.70 |  |
| **Men** | 5.06±0.46  p<0.001 | 5.91±0.32  p=0.057 | 5.06±0.33  p=0.012 | 6.01±0.36  p=0.202 | 7.92±2.84  p<0.001 | 9.30±3.70  p=0.429 | p<0.001 |
| **1-hPG (mmol/L)** | | | | | | | |
| **Women** | 8.07±2.04 | 8.93±2.23 | 9.72±2.08 | 10.86±2.24 | 12.06±3.34 | 11.63±3.57 |  |
| **Men** | 8.03±2.08  p=0.266 | 9.34±2.55  p<0.001 | 9.94±2.08  p=0.027 | 11.38±2.21  p<0.001 | 11.89±3.10  p=0.628 | 12.05±3.10  p=0.505 | p<0.001 |
| **2-hPG (mmol/L)** | | | | | | | |
| **Women** | 6.19±1.19 | 6.31±0.98 | 8.84±0.89 | 9.02±0.90 | 12.41±2.78 | 10.45±3.68 |  |
| **Men** | 5.58±1.21  p<0.001 | 5.88±1.08  p<0.001 | 8.84±0.90  p=0.947 | 8.98±0.90  p=0.372 | 12.15±2.79  p=0.195 | 9.82±3.81  p=0.168 | p<0.001 |
| **HbA_1c_ (%) (mmol/mol)** | | | | | | | |
| **Women** | 5.4±0.4 (35.8±4.8) | 5.6±0.3 (37.2±3.6) | 5.5±0.4 (36.5±4.3) | 5.7±0.4 (38.8±4.0) | 6.3±1.3 (44.9±14.7) | 7.8±2.2 (62.0±24.4) |  |
| **Men** | 5.4±0.4 (35.4±4.5)  p<0.001 | 5.5±0.4 (37.0±4.3)  p=0.100 | 5.5±0.3 (36.5±3.4)  p=0.924 | 5.7±0.4 (38.9±4.3)  p=0.826 | 6.6±1.7 (48.7±19.0)  p<0.001 | 8.1±2.4 (64.7±25.8)  p=0.005 | p<0.001 |
| **Serum creatinine (mmol/L)** | | | | | | | |
| **Women** | 0·064±0·011 | 0·065±0·010 | 0·064±0·011 | 0·065±0·011 | 0·065±0·012 | 0·066±0·015 |  |
| **Men** | 0·081±0·015  p<0·001 | 0·083±0·013  p<0·001 | 0·083±0·014  p<0·001 | 0·085±0·015  p<0·001 | 0·084±0·019  p<0·001 | 0·085±0·020  p<0·001 | p=0.044 |
| **Fasting insulin (pmol/L)** | | | | | | | |
| **Women** | 51·4±46·5 | 61·8±45·1 | 51·4±50·7 | 65·3±41·7 | 84·7±86·8 | 70·8±67·4 |  |
| **Men** | 50·7±52·8  p=0·522 | 61·8±59·0  p=0·863 | 52·1±58·3  p=0·689 | 66·7±58·3  p=0·600 | 92·4±107·0  p=0·179 | 64·6±75·7  p=0·827 | p<0.001 |
| **HOMA-IR** | | | | | | | |
| **Women** | 1.6±2.2 | 2.1±1.9 | 1.7±2.9 | 2.3±1.8 | 3.8±5.2 | 4.1±4.5 |  |
| **Men** | 1.6±2.5  p=0.838 | 2.1±2.4  p=0.556 | 1.7±2.6  p=0.903 | 2.4±2.7  p=0.365 | 4.5±6.8  p=0.036 | 3.8±4.9  p=0.635 | p<0.001 |
| **hsCRP (mg/L)** | | | | | | | |
| **Women** | 3.2±4.9 | 3.8±6.7 | 3.9±5.9 | 4.8±8.3 | 5.4±7.1 | 6.1±9.7 |  |
| **Men** | 2.9±6.1  p=0.011 | 3.6±7.8  p=0.220 | 4.1±9.6  p=0.706 | 4.8±9.4  p=0.813 | 5.8±14.0  p=0.543 | 4.5±11.5  p=0.001 | p=0.014 |
| **Triglycerides (mmol/L)** | | | | | | | |
| **Women** | 1·27±0·78 | 1·35±0·76 | 1·44±0·78 | 1·57±0·83 | 1·78±1·12 | 1·87±1·24 |  |
| **Men** | 1·58±0·93  p<0·001 | 1·71±1·55  p<0·001 | 1·63±0·88  p<0·001 | 1·86±1·10  p<0·001 | 2·06±1·69  p<0·001 | 2·15±1·72  p<0·001 | p<0.001 |
| **HDL-cholesterol (mmol/L)** | | | | | | | |
| **Women** | 1·32±0·32 | 1·28±0·32 | 1·30±0·33 | 1·25±0·32 | 1·23±0·32 | 1·22±0·31 |  |
| **Men** | 1·09±0·26  p<0·001 | 1·08±0·26  p<0·001 | 1·12±0·33  p<0·001 | 1·01±0·29  p<0·001 | 1·04±0·26  p<0·001 | 1·04±0·25  p<0·001 | p=0.055 |
| **NonHDL-cholesterol (mmol/L)** | | | | | | | |
| **Women** | 3·35±0·98 | 3·53±1·01 | 3·64±1·01 | 3·80±0·99 | 3·95±1·09 | 3·91±1·14 |  |
| **Men** | 3·60±0·98  p<0·001 | 3·74±1·02  p=0·011 | 3·80±0·94  p=0054 | 3·98±1·16  p=0·251 | 3·97±1·16  p=0·200 | 3·85±1·15  p<0·001 | p=0.247 |
| GT, glucose tolerance; IFG, impaired fasting glucose; IGT, impaired glucose tolerance; DM, diabetes mellitus; sBP, systolic blood pressure; dBP, diastolic blood pressure; FPG, fasting plasma glucose; 1-hPG, 1-hour plasma glucose; 2-hPG, 2-hour plasma glucose; HbA_1c_, glycated hemoglobin A_1c_; eAG, estimated average glucose; eGFR-MDRD, estimated glomerular filtration rate derived from ‘Modification of Diet in Renal Disease’ study; HOMA-IR, Homeostasis model assessment -insulin resistance; hsCRP, high sensitive C-reactive protein; HDL-cholesterol, high density lipoprotein-cholesterol.  *Frequencies are given as ‘mean (95% CI); all others are given as ‘mean ± SD’, and p values for Women vs. Men. | | | | | | | |
